# Converging metabolic and functional networks for tremor expression and deep brain stimulation-mediated control

**DOI:** 10.1038/s41531-026-01388-7

**Published:** 2026-05-20

**Authors:** Benedikt Weigl, Regina Pistorius, Joachim Brumberg, Nicoló G. Pozzi, Andreas Buck, Muthuraman Muthuraman, Ioannis U. Isaias, Jens Volkmann, Juho Joutsa, Martin M. Reich

**Affiliations:** 1 Department of Neurology, University Hospital and Julius-Maximilian-University Würzburg, Würzburg, Germany; 2 Department of Nuclear Medicine, Medical Center – University of Freiburg, Freiburg, Germany; 3 Department of Nuclear Medicine, University Hospital and Julius-Maximilian-University Würzburg, Würzburg, Germany; 4 Informatics for Medical Technology, Institute of Computer Science, University Augsburg, Augsburg, Germany; 5 Parkinson Institute, ASST Gaetano Pini-CTO, Milan, Italy; 6 Turku Brain and Mind Center, Clinical Neurosciences, University of Turku, Turku, Finland; 7 Turku PET Center, Neurocenter, Turku University Hospital, Turku, Finland

**Keywords:** Diseases, Neurology, Neuroscience

## Abstract

Emerging evidence indicates that movement disorders arise from symptom-specific rather than disease-specific brain network dysfunctions that can be influenced through targeted neuromodulation. Such networks are widely mapped using normative connectome analyses from lesion and stimulation sites. Here, we used [^18^F]-fluorodeoxyglucose (FDG)-PET in 14 essential tremor patients undergoing thalamic deep brain stimulation (DBS) to identify stimulation-induced and tremor related regional metabolic changes in a within-subject design and combined this with normative connectome results. Stimulation increased metabolism in motor cortical and cerebellar regions - key hubs of the previously proposed tremor treatment network as derived from normative functional connectivity. Importantly, individual alignment with this network predicted clinical tremor improvement (*R*^*2*^ = 0.593, *p* = 0.007). These same regions showed higher metabolism during tremor expression in the untreated condition, suggesting overlap between the circuits involved in symptom generation and therapeutic response. These findings support indirect connectome-based models by linking them to brain glucose metabolism changes and suggest that DBS relieves tremor by modulating the same circuit that underlies symptom expression.

## Introduction

Movement disorders are increasingly understood as symptom- rather than disease-specific brain network disorders^1,2^. This paradigm shift has been driven by advances in normative connectomics to disentangle network effects of brain lesions^3–6^, DBS^7,8^, or transcranial magnetic stimulation^9^.

Recently, this approach has been used to identify a common brain network for tremor treatment^2^ integrating results from concurrent EMG-fMRI^10^, brain atrophy patterns^11^, tremor-relieving brain lesions^5^, and DBS targets across different diseases (thalamus in essential tremor (ET) and the subthalamic nucleus and globus pallidus internus in Parkinson’s disease)^7,8,12,13^. Connectivity between neuromodulation targets or beneficial lesions and a symptom-specific network, which includes the primary motor cortex (M1) and cerebellar lobules V, VI, and VIIIa, was associated with tremor relief in a control cohort^2^.

Although prior studies support the clinical translation of indirect fMRI-based normative connectomic models^14,15^, these computational approaches remain correlational and may not fully capture individual- or disease-specific pathophysiology^5,16,17^. Incorporating complementary imaging modalities is essential to draw mechanistic inferences and validate emerging network models^18^. [^18^F]fluorodeoxyglucose (FDG)-PET imaging offers the unique opportunity to study such network and stimulation effects by measuring actual changes in metabolic uptake^19–21^, but direct comparison and integration with findings from normative computer models are lacking.

Here, we integrate normative functional connectivity and stimulation-induced metabolic change by analysing brain glucose metabolism using FDG-PET in essential tremor patients treated with thalamic DBS in a within-subject design.

## Results

### DBS-induced Tremor reduction and metabolic changes

The FDG-PET analysis of tremor relief in 14 tremor patients treated with bilateral thalamic DBS followed the entry criteria, acquisition and processing of our previous work^21^. Patients were evaluated in two conditions: Stim-On (clinically optimized DBS settings) and Stim-Off (DBS switched off for ≥ 72 h). In each condition patients underwent clinical testing with the Fahn-Tolosa-Marin Tremor Rating Scale (FTM-TRS) items 1-9 and an FDG-PET scan (Supplementary Table 1).

**Fig. 1:**
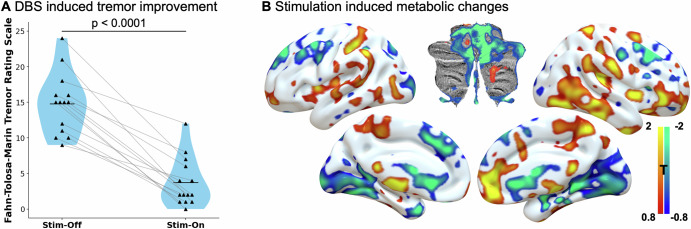
Stimulation-induced clinical and metabolic changes. **A** Stimulation induced tremor improvement: *n* = 14 patients with essential tremor and deep brain stimulation of the (sub) thalamic area assessed using the FTM Tremor Rating Scale. Tremor improved significantly by a mean of 75% (Paired T-Test p = 3.23 × 10⁻⁷) from Stim-Off: 14.79 ± 1.14 to TRS Stim-On: 3.71 ± 0.91. **B** Group-level T-map of stimulation-induced metabolic changes: paired T-Test of FDG-PET scans in Stim-On and Stim-Off condition. Areas of increased/decreased metabolic uptake in red/blue. Increased uptake centred around the stimulation site, the motor cortex and the cerebellar white matter including the dentate nuclei, whereas reduced uptake was observed in the cerebellar cortex, occipital lobes, and frontal cortex.

Comparison of the clinical stimulation effect using a paired t-test between Stim-On vs. Stim-Off Tremor scores revealed a highly significant reduction in tremor severity (FTM-TRS mean ± SEM: Stim-Off 14.79 ± 1.14; Stim-On 3.71 ± 0.91), with an average relative tremor reduction of 75% (Range 33–100%) (absolute change: -11.07 ± 1.16; *p* = 3.23 × 10^−7^) (Fig. 1A).

Stimulation-induced changes in glucose metabolism analysed using a voxel-wise one sample t-tests of the subtraction images (On-Off) using FSL-PALM^22^ revealed increased metabolic uptake around the thalamic stimulation area, in bilateral connected motor network nodes (including supplementary motor area (SMA), primary motor cortex (M1), and the cerebellar white matter including the dentate nuclei). Relatively decreased uptake was observed in occipital and frontal regions and the cerebellar cortex (Fig. 1B). These hubs are similar to the ones implicated in the tremor treatment network published by Goede et al., with the notable addition of increased metabolism at the stimulation site.

A similar pattern was identified when instead using a multivariate spatial covariance analysis (OrT/CVA), which showed strong visual overlap with the voxel-wise paired t-map, corroborated by statistically significant spatial similarity in a Moran spectral randomization analysis (*p* = 0.006; *p* = 0.002; Supplementary Fig. 4).

### Network metabolism outperforms local changes in predicting clinical outcome

**Fig. 2:**
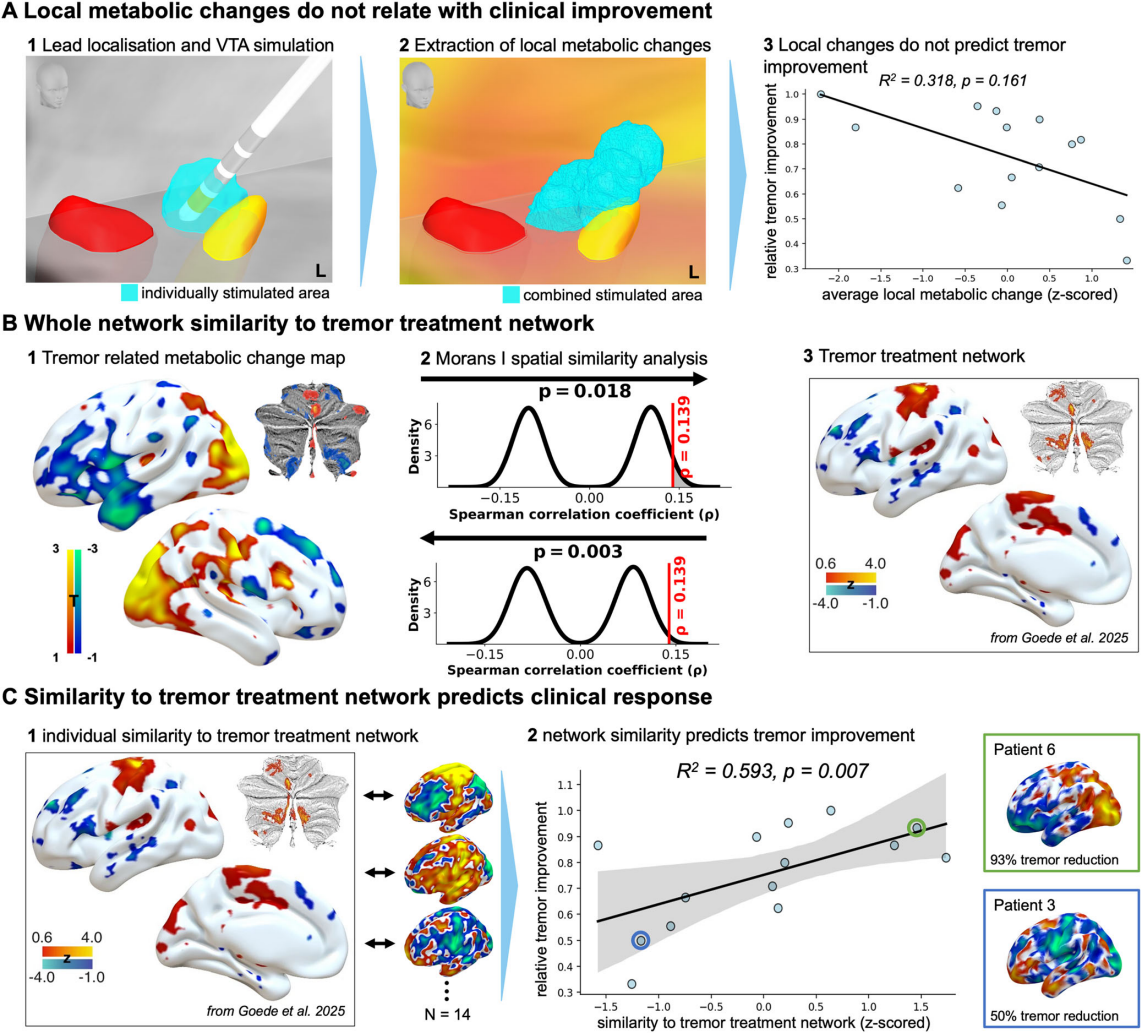
Linking metabolic changes and clinical outcome. **A** Metabolic changes within the combined stimulation area (defined as overlap of ≥3 VTAs) do not relate with clinical outcome when controlling for stimulation amplitude in a multiple linear regression. **B** Whole-brain spatial network similarity analysis using Moran’s I and 10,000 spatial surrogates comparing the stimulation-induced metabolic change map and the unified tremor treatment network. Map at arrow origin is permuted; compared to unpermuted target map. Network maps are spatially significantly more alike than expected by chance. **C** The degree of similarity between an individual patient’s metabolic changes and the published tremor treatment network strongly relates with clinical tremor improvement in a linear regression model controlling for amplitude *R*² = 0.593, *p* < 0.007. Shown are two examples (patients 6 and 3), illustrating FDG-PET changes alongside their respective tremor improvement scores. Network similarity was quantified by a voxel-wise spearman rank correlation. **B3** and **C1** reproduced from Goede et al. ^2^, licensed under CC BY 4.0 (https://creativecommons.org/licenses/by/4.0/).

FDG uptake in the general stimulation area ( ≥ 3-VTA overlap) was associated with stimulation amplitude (R² = 0.628, *p* = 0.001), consistent with previous studies^23,24^. Beyond amplitude, pulse width and frequency showed no independent effects on local FDG uptake (Supplementary Table 2). A weaker, negative association was observed with increased metabolic uptake and tremor improvement (*R²* = 0.316, *p* = 0.036), which however became non-significant when controlling for amplitude (*R²* = 0.318, *p* = 0.161). Results were near identical when FDG uptake was taken from patients individual VTAs (Supplementary Fig. 1). In other words, local metabolic changes at the stimulation site, although related to stimulation amplitude, could not be clearly linked to tremor outcome (Fig. 2A).

Beyond local effects, the group-level tremor-related metabolic network change showed significant spatial similarity to the published bilateral tremor treatment network^2^. The voxel-wise Spearman rank correlation between both networks significantly outperformed 10,000 spatially autocorrelated surrogate maps generated using Moran’s spectral randomization (*p* = 0.018 and *p* = 0.003 – both maps permuted in turn; Fig. 2B)^25,26^. At the individual level, alignment with the tremor treatment network was not associated with any stimulation parameter (Supplementary Table 2), but was significantly associated with greater tremor improvement (*R²* = 0.364, *p* = 0.022). Importantly, unlike the local effects this association remained robust, even when including the stimulation amplitude as a covariate (*R²* = 0.593, *p* = 0.007; Fig. 2C). Similar results were obtained when using the sum of a voxel-wise multiplication as an alternative network similarity metric or when averaging PET maps to the left hemisphere to match the lateralization of the original published treatment network^2^ (Supplementary Fig. 2).

In summary, on the group-level the tremor related metabolic change map showed greater spatial similarity to the tremor treatment network derived from normative functional connectivity than expected by chance, and individual network alignment predicted tremor suppression independent of stimulation amplitude.

### Modulation of the tremor network

**Fig. 3:**
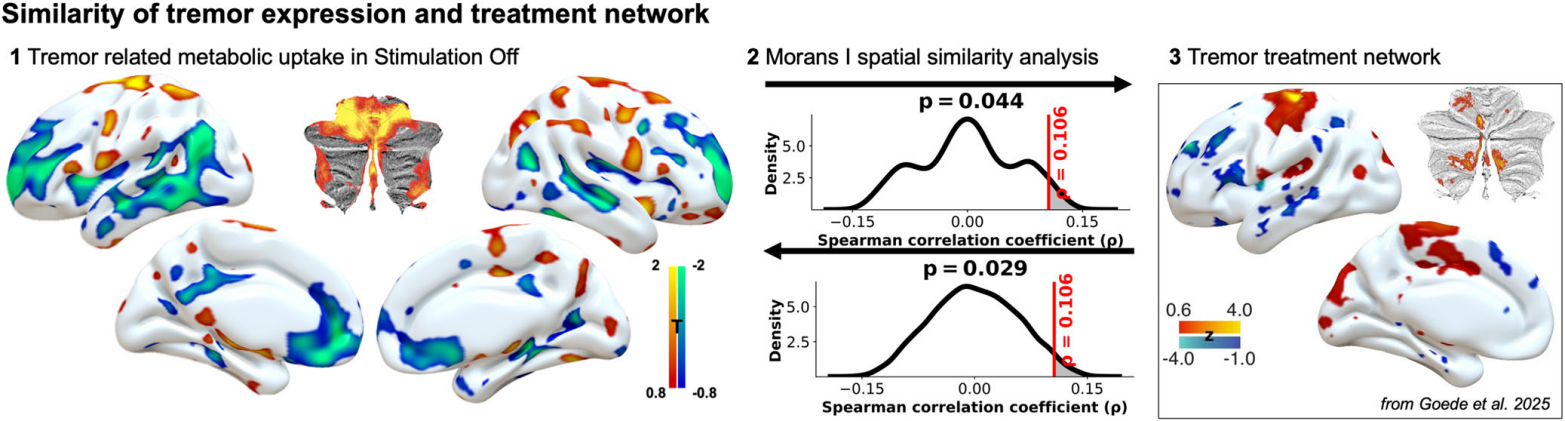
Similarity between tremor expression and treatment network. Whole-brain network similarity analysis using Moran’s I and 10,000 spatial surrogates to compare the spatial alignment between the network indicating tremor expression related metabolic uptake in the Stim-Off condition and the unified tremor treatment network (Goede et al.). Map at arrow origin is permuted; compared to unpermuted target map. Network maps are significantly more alike than expected by chance, indicating a high similarity. Panel 3 reproduced from Goede et al. ^2^, licensed under CC BY 4.0 (https://creativecommons.org/licenses/by/4.0/).

A tremor-expression–related metabolic network was derived using voxel-wise linear regression between Stim-Off FDG uptake and baseline tremor severity. Higher tremor severity was associated with increased metabolism in the cerebellum and motor cortex, and with decreased metabolism in frontal and temporal regions. Interestingly, this closely resembled the reported network involved in the treatment of tremor^2^. The network spatial similarity analysis using Moran’s I and 10,000 spatial surrogates confirmed that this overlap was greater than expected by chance (*p* = 0.044 and *p* = 0.029), indicating that effective treatment targets the same network involved in tremor expression (Fig. 3).

A corresponding regression of residual tremor scores in the Stim-On condition, conducted as a complementary analysis, revealed a highly similar tremor-related metabolic pattern (whole-brain MSR, *p* < 0.0001), though with relatively greater uptake in the supplementary motor area, motor cortex, and the region surrounding the stimulation site (Supplementary Fig. 5). Consistent with the stimulation shaping the metabolic pattern in this Stim-On condition, this pattern also showed greater-than-expected similarity to the On–Off metabolic stimulation effect (whole-brain MSR, *p* = 0.038 and *p* = 0.026), whereas no such similarity was observed for the Stim-Off tremor expression map (whole-brain MSR, *p* = 0.620 and *p* = 0.609).

### Modelling metabolic changes using stimulation features

**Fig. 4:**
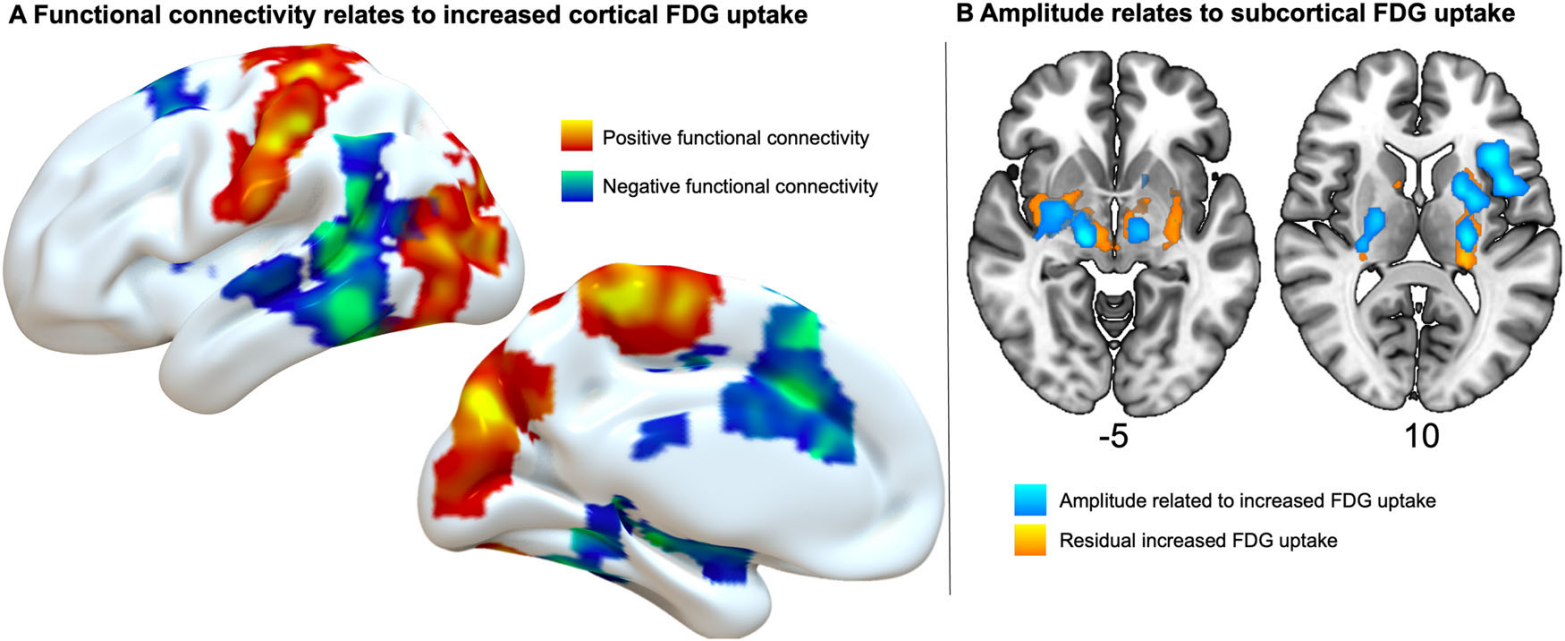
Metabolic changes influenced by stimulation features in mixed linear model. Voxel-wise mixed linear model predicting FDG-PET changes using individual positive and negative normative functional connectivity maps and demeaned stimulation amplitude as covariates. T-Maps of RFT-FEW corrected significant clusters (cluster-*p*-value < 0.05). **A** Surface projections of positive (red-yellow) and negative (blue-green) connectivity with the stimulation site were each linked to increased metabolism in cortical regions including the motor/occipital and temporal regions, respectively. **B** Representative cross slices, of stimulation amplitude (light blue) was associated with subcortical uptake near the stimulation site, while notable additional residual effects, not explained by either connectivity or amplitude, were centred in the basal ganglia (yellow).

Finally, we used the VoxelStats^27^ pipeline in an exploratory analysis modeling each patient’s metabolic changes as a function of individual voxel-wise normative connectivity and stimulation amplitude. Increased positive (correlated) connectivity was associated with increased metabolic activity in the cortical motor area and occipital cortex (red-yellow). In contrast, negative (anticorrelated) connectivity was linked to increased uptake in the temporal lobe (blue-green; Fig. 4A). Both directions of normative functional connectivity were not significantly associated with decreased cortical FDG uptake. Stimulation amplitude, as expected, showed a positive association with FDG uptake near the stimulation field (light-blue). Additional residual metabolic changes not explained by either amplitude or connectivity were observed as increased uptake centred in the basal ganglia (yellow; Fig. 4B). For both connectivity directions, FWE corrected significant clusters were observed in the left hemisphere only, although qualitatively similar, albeit non-significant patterns were evident in the right hemisphere (Supplementary Fig. 3).

## Discussion

While normative connectomics have improved our understanding of brain-wide network dynamics, direct metabolic validation remains limited. Here, we provide such evidence using a within-subject, two-condition FDG-PET design to map tremor-related and stimulation-induced metabolic changes in thalamic DBS for essential tremor. By integrating this data with a recently published normative fMRI-based tremor treatment network and individual normative functional connectivity, we link functional connectivity from the stimulation site and metabolic response. Four main findings emerge: (i) local metabolic effects are not linked to clinical outcome, (ii) normative connectivity and metabolic changes converge in a symptom-specific brain-wide network predictive of tremor relief across diseases, (iii) the networks involved in tremor expression and tremor relief are closely aligned (iv) exploratory modelling suggests that stimulation induced cortical metabolic changes are shaped by functional connectivity to the stimulation site.

DBS increased local metabolic uptake near the stimulation site with rising amplitude, consistent with prior findings^15,21,23^. However, these local effects showed no consistent relationship with tremor outcome. In contrast, network similarity to the functional connectivity defined tremor-treatment network predicted tremor relief, independent of stimulation amplitude. These diverging effects were further evidenced by the exploratory whole-brain linear model, where increased cortical metabolism was associated with stronger functional connectivity to the stimulation site while amplitude-related effects were spatially confined to the vicinity of the lead.

The inverse, amplitude-driven relationship between local metabolic activity and tremor relief challenges the notion that symptom improvement results from local inhibition alone. Instead, the amplitude-independent network-level effects support the view that effective tremor-relieving DBS engages a distributed network through connectivity-dependent mechanisms, rather than acting via focal current spread^2^. Residual changes in the basal ganglia observed in the linear model were not accounted for by either connectivity or amplitude and may reflect upstream modulation, disease-specific contributions, or compensatory processes that warrant further investigation^28^.

Stimulation-induced metabolic changes centred on the bilateral motor cortex and cerebellar white matter, and aligned with prior reports of stimulation-induced increased FDG-uptake in the dentate nuclei and reported BOLD signal increases in the motor cortex in ET specifically^29,30^. These changes showed high spatial similarity with the tremor treatment network derived from normative functional connectivity^2^. The network also mirrors the one reported in a comparable FDG-PET study of thalamic DBS in Parkinson’s disease (PD) related to tremor, which also implicated the motor cerebellum, dentate nucleus, and motor cortex in tremor^24^.

These hubs consistently involved in effective tremor-relieving DBS, showed a high spatial alignment with tremor-related metabolic changes in unstimulated patients and further correspond to the cerebello-thalamo-cortical circuit proposed as a shared expression pathway in the “dimmer-switch” model of tremor generation^31–33^. In this model distinct “switches” such as the basal ganglia or thalamus in PD^31^, or the cerebellum in ET^18^, are thought to initiate disease-specific pathological signals, which then act through a common tremor expression network^34^. Together with additional tremor modulators such as the locus coeruleus^35,36^, this model would explain both the clinically observed heterogeneity as well as the anatomical convergence of affected regions across tremor syndromes. Tremor treatment thus appears to act by modulating the circuit involved in tremor expression a pattern also reflected in the residual tremor-related metabolic profile during active stimulation.

Although PET cannot distinguish direct from compensatory effects^37^, and neither PET nor normative connectivity can establish causality per se, the convergence of spatial patterns across imaging modalities strengthens the inference of a targeted modulation of this network. Notably, regions connected to the stimulation site regardless of direction (positive or negative), exhibited increases in metabolism with no significant decreases observed. This observation aligns with the idea that both connectivity directions (positive and negative) capture different kinds of functional coupling via BOLD synchrony^16,38^. Despite reflecting distinct underlying mechanisms^39^, both types can therefore plausibly drive increased neuronal and metabolic activity. In contrast, regions lacking connectivity would be expected to show minimal metabolic response, and thus any metabolic changes there may rather point towards compensatory effects. However, caution is warranted, as the interpretation of resting-state anticorrelations remains a matter of debate^40^.

Taken together, the cross-disorder and cross-modal spatial convergence of stimulation effects lends support to the hypothesis that DBS acts directly on this tremor expression network, rather than via compensatory mechanisms^1,2,23,41^. More broadly, these findings highlight the added value of multimodal approaches for dissecting DBS mechanisms, and further support the utility of normative connectivity for exploratory analyses in the absence of patient-specific data^8,16^.

Interestingly however, despite the anatomical convergence, tremor-relieving DBS in PD was associated with reduced tremor-network metabolism contrasting our observed cortical increases in ET. While this divergence might partly reflect rebound effects due to different stimulation washout protocols^21,24^, it could indicate disease-specific pathophysiology and modulation effects within the shared tremor network^34^. In a previous study, DBS-related increases in motor cortex metabolism in ET were found to reverse disease-related reductions relative to healthy controls leading to the hypothesis of compensatory activation^42^. However, the tremor-related hypermetabolism we observed in the unstimulated state, in line with similar reports in a recent FDG-PET study^43^, challenges this interpretation, as it suggests a direct involvement in symptom expression^31–33^. Furthermore, the fact that these metabolic changes are functionally connected to the stimulation site makes a nonspecific compensatory response less likely and instead supports a mechanistic link and targeted network modulation of the tremor circuit.

We therefore hypothesize that DBS alleviates tremor by disrupting the propagation of pathological activity via the modulation of the cerebello-thalamo-cortical tremor circuit, while preserving or restoring physiological signalling. This is supported by the observed correlation between network-level metabolic changes and clinical improvement alongside the convergence of these effects with normative functional connectivity to the stimulation site. It also aligns with proposed DBS mechanisms involving regularization of neural firing across the cortico-basal ganglia-thalamo-cortical circuit^44^. This could also explain the increased metabolic uptake in the motor cortex during successful DBS, which may reflect a stimulation-related shift to a therapeutically relevant network state rather than simply returning it to a pre-pathological one^44^. While the cross-disease convergens reinforces the concept of symptom-specific rather than disease-specific networks being modulated across disorders^41,45,46^, the divergent pathological and modulatory effects across diseases within this network could imply that the pathology- or stimulation-related functional brain states of these shared circuits are disease-specific^34^.

A key limitation is our focus on essential tremor, possibly limiting generalizability. However, as shown, the identified network hubs align with those found across different tremor syndromes, modalities and therapeutic targets^2,24^. While our sample size (*N* = 14) is modest, it exceeds typical within-subject metabolic neuroimaging cohorts^47^, and the hypothesis-free, whole-brain, within-patient-controlled analysis adds confidence to the findings. Additionally, as a 72 h washout is required to avoid residual stimulation effects influencing the Off condition^21,29^, nonspecific between-session influences cannot be fully excluded. Though the paired within-subject design helps mitigate their impact at the group level. A potential clinical confound may underlie the relationship between stimulation amplitude, local metabolic uptake, and tremor outcome, as higher amplitudes may reflect attempts to treat more resistant tremor. Nevertheless, the lack of association between amplitude and network-level effects suggests that this does not compromise our conclusions regarding network mechanisms. Furthermore, the univariate voxel-wise approaches used have known limitations regarding covariance structure and voxel-wise inference, but remain well suited for spatial localization of metabolic effects^48^, and the main stimulation-associated spatial pattern was corroborated by the exploratory multivariate approach (OrT/CVA). Finally, our exploratory linear model should be interpreted with caution due to the artificial separation of positive and negative connectivity values, which may have introduced shifted voxel-wise distributions. However, directional separation of predictors has been necessary in neuroimaging to examine divergent functional effects, and this analysis serves an initial step to explore the relationship between connectivity, amplitude, and metabolism^49,50^.

In conclusion, our FDG-PET findings provide direct metabolic evidence supporting the proposed tremor treatment network and emphasize the relevance of engaging these network hubs in ET patients undergoing thalamic DBS, bridging the findings from an indirect connectome approach of large cohorts to within-subject measurements in individual patients. Importantly, the same circuit implicated in tremor expression in the “dimmer-switch model” appears to be modulated by DBS in ET, suggesting that therapeutic efficacy relies on modulation of this symptom-specific network. Continued methodological development, particularly in multimodal data integration, together with broader clinical validation, will be crucial to fully characterize the underlying network dynamics.

## Methods

### Participants and clinical testing

Fourteen patients with a clinical diagnosis of essential tremor (ET)^51^ were recruited (7 males; mean age 64 ± 4 years; mean disease duration 27 ± 4 years; mean time since DBS implantation 32 ± 5 months; mean ± SEM). All patients were treated with bilateral thalamic DBS implanted following the surgical procedure described by Herzog et al. ^52^, showed a stable therapeutic response ( > six months) having discontinued any anti-tremor medications at the time of evaluation. Patients were evaluated in two conditions: Stim-On, using their clinically optimized DBS settings, and Stim-Off, following ≥72 h of DBS discontinuation. In each condition, patients underwent clinical assessment using items 1–9 of the Fahn-Tolosa-Marin Tremor Rating Scale (FTM-TRS) and a resting-state FDG-PET scan (Supplementary Table 1).

### Ethics approval

The study was conducted in accordance with the Declaration of Helsinki and approved by the institutional review board of the University Hospital of Würzburg (Aktenzeichen: 283/14) and the Governmental Radiation Protection Authority (Bundesamt für Strahlenschutz, Aktenzeichen: Z5-22463/2-2015-010). Written informed consent was obtained from all participants.

### FDG-PET Imaging, preprocessing and functional connectivity estimation

PET scans were acquired on a Biograph mCT 64 scanner (Siemens). After overnight fasting, patients received 207 ± 12.9 MBq of FDG, with the subjects’ eyes open in a dimly lit room and with minimal auditory stimulation. Imaging began 30 min post-injection (3D mode, single 10 min bed position,). Low-dose CT provided attenuation correction. Images were reconstructed iteratively (24 subsets, three iterations, 400×400 matrix, Gaussian filtering, ~2 mm axial resolution). Data preprocessing (SPM8, Wellcome Department of Cognitive Neurology, University College, London) included spatial normalization to an FDG template in MNI space^53^, intensity normalization using a brain parenchyma mask, and smoothing with an 8 mm Gaussian kernel following our previously established pipeline^21^. Local Volumes of tissue activated (VTAs) were estimated from the individual stimulation settings using the SimBio/FieldTrip VTA model implemented in Lead-DBS (lead-dbs.org), with otherwise standard settings^8,54^. The combined bilateral VTAs were then used as seed regions in the Lead-Mapper submodule to estimate normative functional connectivity profiles. For Patient 2 (interleaving stimulation), both VTAs of each hemisphere were merged.

### Statistical analysis

Clinical tremor improvement was analysed using a paired t-test between the tremor scores in Stim-Off and Stim-On conditions. To assess tremor-related baseline metabolism, we performed a voxel-wise linear regression of the Stim-Off PET images against individual tremor severity scores, with an additional regression using to Stim-On images and residual tremor scores. Stimulation-induced metabolic changes were assessed by subtracting the Stim-Off image from the Stim-On image for each subject (On–Off). General stimulation effects were analysed using a voxel-wise one sample t-test on these difference images, while tremor improvement–related changes were assessed using a voxel-wise linear regression with relative tremor improvement scores. All voxel-wise analyses were performed using FSL-PALM^22^ and cerebellar flatmaps were generated using the SUIT toolbox^55^.

To cross-validate the univariate findings, we performed an Ordinal Trends Canonical Variates Analysis between both stimulation conditions (OrT/CVA; software available at http://groups.google.com/group/gcva) using a simplified model restricted a priori to the first five principal components. Voxel-weight stability was assessed by bootstrap resampling (100 iterations), and the resulting components were linearly combined into a general spatial covariance pattern^24,56,57^.

Spatial similarity between both group-level patterns was evaluated using a voxel-wise Spearman rank correlation with significance estimated via Moran’s spectral randomization (MSR) implemented in the BrainSpace pipeline^25,26^. By leveraging eigenvectors of a spatial weight matrix, MSR enables comparison of the observed correlations against a null distribution of spatially similar surrogate networks. We used inverse squared inter-voxel distance as weights to avoid arbitrary distance thresholds and reduce overweighting of distant voxels. For each comparison, 10,000 surrogate maps were generated, and p-values were calculated as the proportion of null correlations equal to or exceeding the observed correlation^58–60^. Networks were down-sampled to 4 mm resolution for this analysis only to allow efficient computation while maintaining spatial structure.

### Local and network effects

To assess the relationship between local stimulation-site metabolism and clinical improvement, we defined a cohort-level general stimulation site as voxels in which at least three patients VTAs overlapped, yielding a common anatomical region identical across patients, following Honkanen et al. ^23^. As a complementary site definition, we also used each patient’s individual VTA. For each stimulation site definition, mean FDG uptake was extracted per subject and z-scored across all values of the cohort for better comparability. Associations between FDG uptake and clinical outcome were tested using Pearson correlation. Stimulation parameters (amplitude, pulse width, and frequency) were screened as potential confounding factors by testing associations with FDG uptake, followed by collinearity assessment and residualized multiple linear regression to isolate independent effects. Parameters with significant independent associations were included as covariates in subsequent models. We next assessed individual-level network similarity to the tremor treatment network defined by Goede et al.^2^. As the published tremor network included only the left hemisphere and cerebellum, the left hemisphere was nonlinearly mirrored to the right to account for the bilateral nature of our PET data using SPM, effectively reversing the nonlinear flip applied in the original publication. For each patient, voxel-wise Spearman correlation between their FDG-PET change map and this non-zero bilateral published tremor treatment network^2^ was used as a similarity metric. This measure was then z-scored and correlated with clinical improvement, screening and controlling for possible confounders, as in the local analysis. To confirm robustness, we repeated the analysis using the sum of voxel-wise multiplications^2^ as alternative similarity metric and by alternatively averaging both PET hemispheres to the left side to match the unilateral treatment network.

### Comparing Tremor expression and treatment networks

We additionally tested whole-group spatial similarity between the tremor-related metabolic FDG network patterns and the bilateral tremor treatment network^2^ using a voxel-wise Spearman rank correlation again assessing significance using MSR. First, we tested whether the stimulation-induced metabolic changes associated with tremor improvement spatially matched the tremor treatment network, and second, whether this treatment network is similar to the network associated with the tremor expression network in Stim-Off patients.

As a supplementary analysis, we further assessed the spatial similarity between the Stim-On residual tremor map and the Stim-Off tremor expression map, and additionally tested both tremor expression maps against the On–Off metabolic stimulation effect, evaluating all three pairwise comparisons using MSR.

### Exploring DBS effects on metabolic activity

In an exploratory analysis, we used VoxelStats^27^ to model each patient’s PET-based metabolic change as a function of voxel-wise normative connectivity and demeaned identified stimulation parameter confounders. As positive or negative connectivity values (correlated or anticorrelated, respectively) likely reflect distinct network effects^39^, we treated them as separate predictors. Individual connectivity maps were split into positive and negative components by zeroing out all voxels of the opposite sign. These predictors were then entered into a general linear mixed model, assessing statistical significance and correcting for family wise error using random field theory cluster-correction as implemented in VoxelStats (FWHM 8 mm, cluster-p-value < 0.05).

## Supplementary information

Supplementary Material

## Data Availability

Final group-level statistical maps are publicly available in a GitHub repository under [https://github.com/BenediktWeigl/Converging-metabolic-and-functional-tremor-networks]. Individual Patient Data are available upon reasonable request pending institutional approval.

## Code availability

Scripts beyond the cited published analysis pipelines are publicly available in the same GitHub repository [https://github.com/BenediktWeigl/Converging-metabolic-and-functional-tremor-networks].
