# Supplementary material for: Converging metabolic and functional networks for tremor expression and deep brain stimulation-mediated control
Source: NPJ Parkinsons Dis. 2026 May 20;12:119. doi: 10.1038/s41531-026-01388-7 (PMC13190717; doi:10.1038/s41531-026-01388-7)
Supplement: Supplementary file 1 — Supplementary Material [file 41531_2026_1388_MOESM1_ESM.pdf]

Supplementary Material

Patient	Sex	Age [yrs]	Disease duration [yrs]	FTM-TRS Stim-OFF/ON	Electrode Type	Amplitude right//left [mA]	Pulsewidth right//left [µs]	Frequency right//left [Hz]	Comment
**1**	M	83	20	12/4	Medtronic 3389	2.3 // 3	30 // 30	180 // 180	
**2**	F	74	12	18/12	Medtronic 3389	3.3/4.5 // 4.5/3.9	30 // 30	120 // 120	interleaving
**3**	M	64	30	16/8	Medtronic 3389	3.9 // 6.3	30 // 30	180 // 180	
**4**	F	72	25	11/2	Medtronic 3389	4.4 // 3.6	30 // 30	130 // 130	
**5**	F	74	50	10/2	Medtronic 3389	3.6 // 3.1	30 // 30	180 // 180	
**6**	M	65	25	15/1	Medtronic 3389	3.9 // 3	30 // 40	125 // 125	
**7**	M	64	50	15/0	Medtronic 3389	2.8 // 1.4	60 // 60	140 // 140	
**8**	F	58	40	10/1	Medtronic 3387	0.9 // 2.5	90 // 60	130 // 130	
**9**	F	20	10	15/2	Medtronic 3387	2.3 // 2	90 // 60	130 // 130	
**10**	M	61	30	16/6	Medtronic 3389	2 // 2.6	60 // 60	170 // 170	
**11**	F	76	11	9/4	Boston Scientific Vercise Cartesia	0.8 // 2	60 // 60	179 // 179	undirected contact
**12**	M	75	20	24/7	Medtronic 3389	3.3 // 4	60 // 60	180 // 180	
**13**	F	59	44	15/2	Medtronic 3389	1.5 // 1.1	60 // 60	180 // 180	
**14**	M	54	10	21/1	Medtronic 3389	2.8 // 2.3	60 // 60	180 // 180	

Supplementary Table 1: Summary of demographic, clinical and stimulation data for the essential tremor cohort. Columns include patient identifiers, general demographics (sex, age at testing and months since DBS) and clinical scores in the Fahn-Tolosa Marin Tremor Rating Scale (FTM-TRS) items 1-9 in both stimulation OFF and ON conditions. The table also reports electrode types and stimulation settings (Amplitude, Pulsewidth and Frequency during the ON condition). Patient 2 received interleaving stimulation. Patient 11 had a directed electrode though the stimulation was set to the undirected lowest contact.

Parameter	Local FDG Uptake (Pearson R)	Collinearity (Pearson R)	Independence	Tremor Network Similarity (Pearson R)
Amplitude	**R = 0.792, p = 0.001**	*(reference covariate)*	*(reference covariate)*	R = 0.090, p = 0.760
Pulse width	**R = -0.584, p = 0.028**	R = -0.740, p = 0.002	No (p = 0.985)	R = 0.078, p = 0.792
Frequency	R = -0.076, p = 0.797	—	—	R = -0.504, p = 0.066

Supplementary Table 2: Stimulation parameter screening for potential confounding effects. Pearson correlations were computed between each stimulation parameter and (i) local FDG uptake at the stimulation site and (ii) individual PET similarity to the tremor treatment network. Both amplitude and pulse width showed significant associations with local FDG uptake. However, pulse width and amplitude were strongly inversely correlated (r = −0.740, p = 0.002), and residualization confirmed that pulse width did not explain variance independent of amplitude (p = 0.985). Frequency showed no significant associations with either measure and was not taken forward. Amplitude was therefore retained as the sole stimulation covariate in all subsequent analyses.

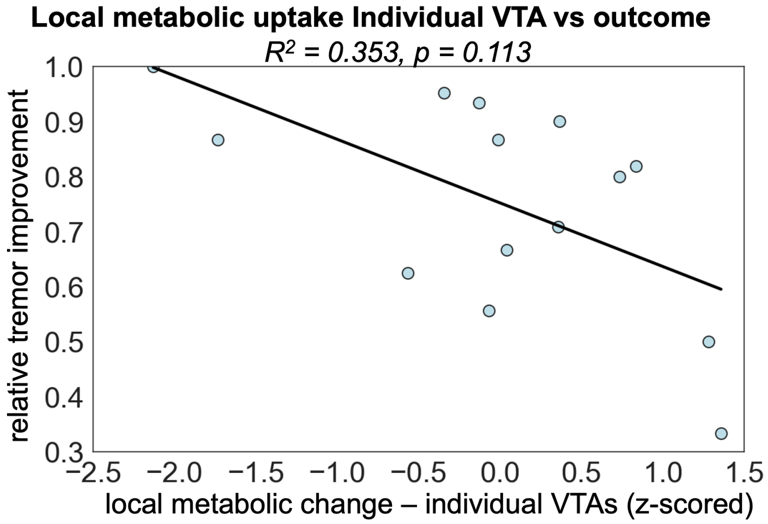

Supplementary Figure 1: Additional local stimulation-site analysis using individually defined VTAs. To complement the cohort-level general stimulation area (≥3-VTA overlap) analysis shown in the main manuscript, we repeated the local stimulation-site analysis using FDG uptake extracted from each patient's individual VTA. The results were near identical: Individual-VTA uptake showed a strong association with stimulation amplitude ($R^2 = 0.511$, $p = 0.004$) and a negative association with tremor improvement ($R^2 = 0.353$, $p = 0.025$) which, consistent with the main analysis, did not remain significant after controlling for stimulation amplitude ($p = 0.113$).

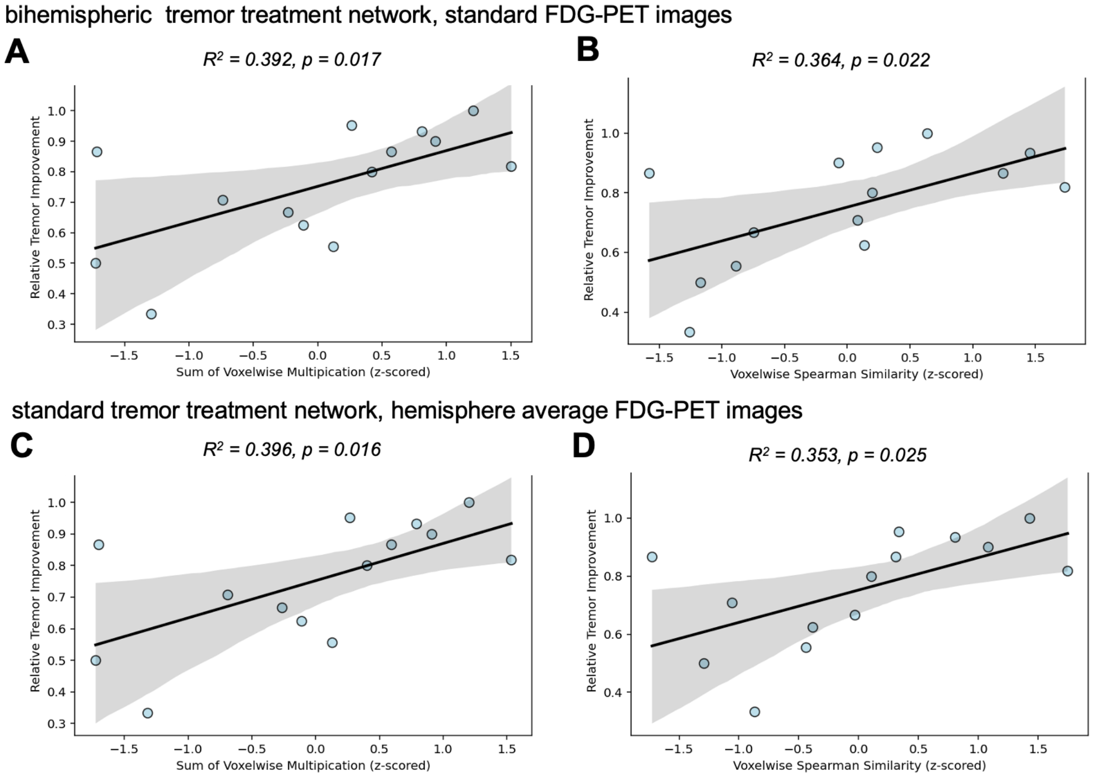

Supplementary Figure 2: Network similarity predicts tremor improvement. Detailed analysis of how individual patients' stimulation-induced metabolic changes align with the convergent tremor treatment network (z-scored) and correlate with clinical tremor improvement.

Top row: The tremor network is mirrored to the right hemisphere and applied to unmodified PET images. Two similarity metrics are shown: **(A)** Sum of Voxel-wise multiplications with the network template, following the method of Goede et al. **(B)** Voxel-wise Spearman correlation between the PET difference image and the network, with both similarity metrics z-scored across all values for comparability. **Bottom row:** The tremor treatment network is left unaltered, and the PET difference images are nonlinearly flipped to the left hemisphere and averaged. Again, two metrics are shown: **(C)** Sum of voxel-wise multiplication. **(D)** Voxel-wise Spearman correlation. Across all methods, similarity to the tremor network showed a consistent and strong positive correlation with clinical tremor improvement. Both voxel-wise similarity measurement-strategies produced nearly identical results.

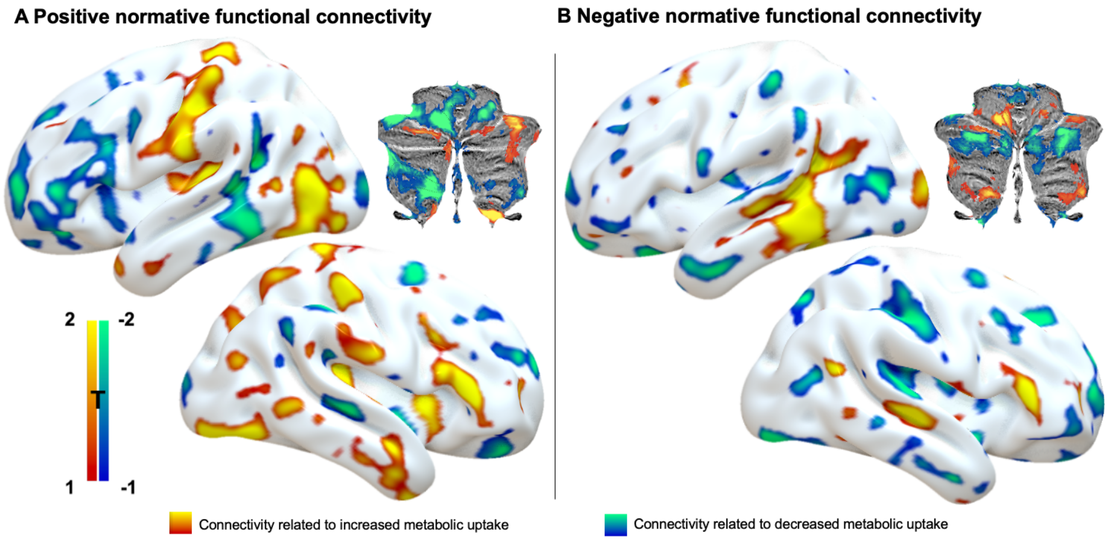

Supplementary Figure 3: Metabolic changes influenced by normative functional connectivity in linear mixed model. Voxel-wise mixed-effects model predicting FDG-PET changes using individual normative functional connectivity. Surface projections show T-maps of a model including positive (A) and negative (B) functional connectivity maps and demeaned stimulation amplitude as covariates. Warm colors (red–yellow) indicate regions where stronger connectivity is associated with increased metabolism; cool colors (green–blue) indicate regions where stronger connectivity is associated with decreased metabolism.

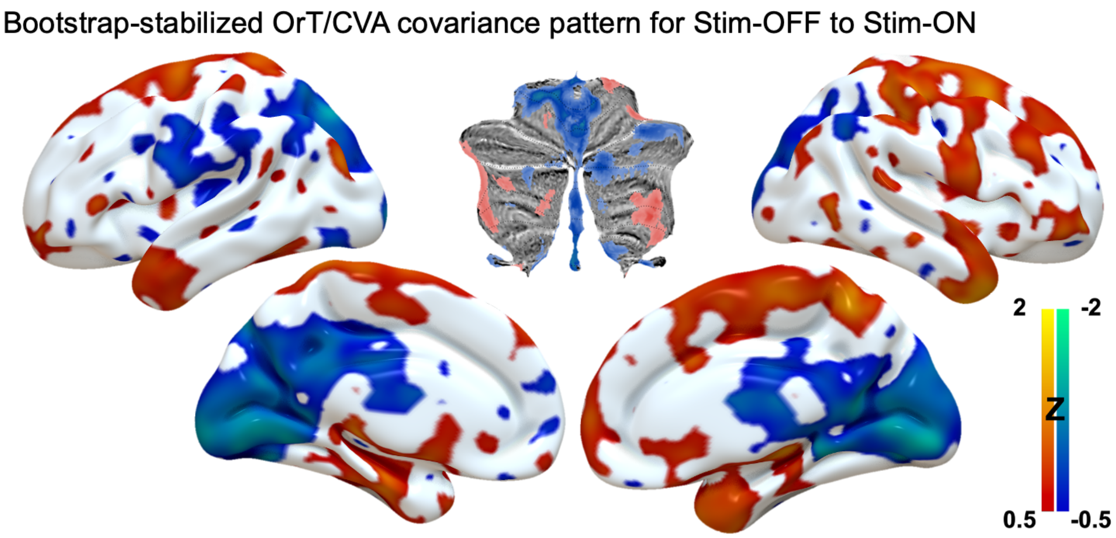

Supplementary Figure 4: Bootstrap-stabilized OrT/CVA spatial covariance pattern contrasting Stim-OFF and Stim-ON. Exploratory OrT/CVA was performed using an a-priori defined model restricted to the first five principal components. The displayed map represents bootstrap-derived Z-scores (100 iterations) of the resulting linearly combined spatial covariance pattern. This multivariate pattern showed high visual overlap with the voxel-wise paired t-map from the main manuscript, which was confirmed by Moran spectral randomization analysis showing greater spatial similarity than expected by chance ($p = 0.006$; $p = 0.002$).

Residual tremor related metabolic uptake Stimulation On

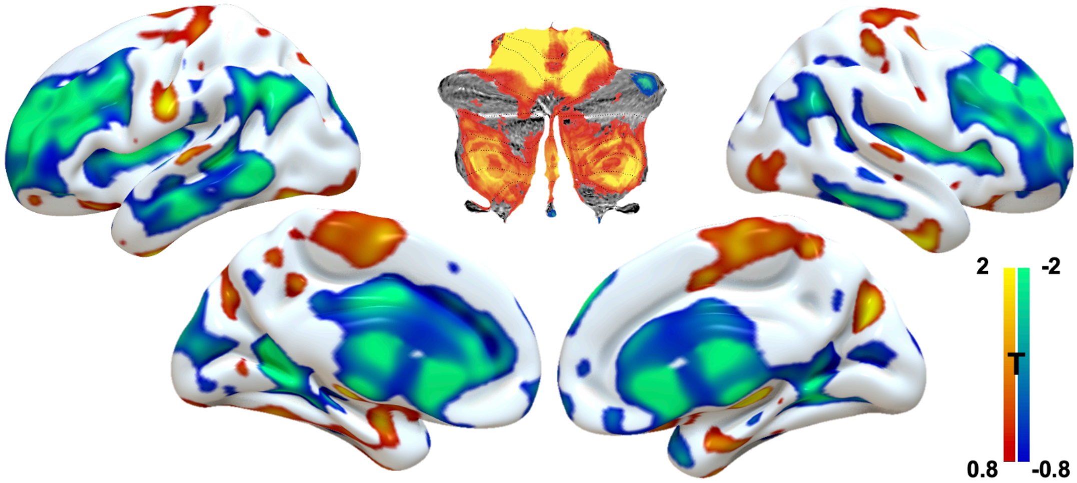

Supplementary Figure 5: Residual tremor-related metabolic pattern in the Stim-ON condition. Voxel-wise regression between tremor severity and FDG uptake during Stim-ON revealed a spatial pattern highly similar to the tremor-related metabolic profile observed in the Stim-OFF condition (whole-brain MSR, $p < 0.0001$ for both directions), while showing relatively greater metabolic uptake in the supplementary motor area, motor cortex, and the region surrounding the stimulation site. The ON–OFF metabolic contrast showed greater-than-expected similarity to the Stim-ON tremor map (whole-brain MSR, $p = 0.038$ and $p = 0.026$), whereas no such similarity was observed for the Stim-OFF tremor map (whole-brain MSR, $p = 0.620$ and $p = 0.609$).
